# Correlation study of fatty pancreas and fatty liver CT manifestations with biochemical parameters in middle-aged and young adult cholecystectomy patients

**DOI:** 10.1186/s12876-025-04447-0

**Published:** 2025-11-20

**Authors:** Yi Zhou, Chao He, Haipeng Zhang

**Affiliations:** 1 Department of Radiology, Shaanxi Provincial Hospital of Traditional Chinese medicine, 4 Ming Palace Road, Lianhu District, Xi’an city, Shaanxi province 710000 China; 2 Department of Radiology, Shaan xi Traffic Hospital, 276 Da Xue South Road, Xi’an city, Shaanxi province 710000 China; 3 Department of General Surgery, Shaanxi Provincial Hospital of Traditional Chinese medicine, 4 Ming Palace Road, Lianhu District, Xi’ancity, Shaanxi province 710000 China

**Keywords:** Cholecystectomy, Pancreatic steatosis, Hepatic steatosis, CT manifestations, Biochemical parameters

## Abstract

**Background:**

To investigate the correlation between the occurrence of pancreatic steatosis, hepatic steatosis, and cholecystectomy, as well as the associated biochemical parameters.

**Methods:**

A retrospective analysis was conducted on 409 patients who underwent abdominal CT scans between November 2022 and March 2024. Among them, 127 patients had undergone cholecystectomy (experimental group), while 282 had not (control group). The incidence rates of pancreatic steatosis and hepatic steatosis were compared between the two groups, along with relevant biochemical parameters including total cholesterol, triglycerides, high-density lipoprotein cholesterol, low-density lipoprotein cholesterol, and fasting blood glucose levels.

**Results:**

The incidence of pancreatic steatosis in the experimental group was 67.8%, and the incidence of hepatic steatosis was 22%. In the control group, the incidence rates were 54.3% for pancreatic steatosis and 15.6% for hepatic steatosis. The rates of both pancreatic and hepatic steatosis significantly increased following cholecystectomy, with pancreatic steatosis showing a notably higher incidence than hepatic steatosis. Statistical analysis revealed a significant correlation between cholecystectomy and pancreatic steatosis (*p* < 0.05), whereas no significant correlation was found with hepatic steatosis.

**Conclusion:**

The incidence rates of both pancreatic and hepatic steatosis are elevated following cholecystectomy, particularly for pancreatic steatosis. The study indicates a significant association between cholecystectomy and pancreatic steatosis. Therefore, it is recommended to closely monitor patients who have undergone cholecystectomy for the development of pancreatic steatosis, allowing for early diagnosis and intervention to prevent the progression of related conditions.

## Introduction

In recent years, with continuous advancements in imaging techniques, research on pancreatic steatosis has garnered increasing attention [1]. Existing studies have found that pancreatic steatosis is closely associated with disorders of glucose metabolism, acute and chronic pancreatitis, and pancreatic malignancies. In routine clinical practice, it is often observed that patients who undergo cholecystectomy exhibit signs of pancreatic steatosis and hepatic steatosis in follow-up abdominal CT scans [2]. However, studies exploring the relationship between cholecystectomy, the incidence of pancreatic and hepatic steatosis, and their associated biochemical parameters are relatively sparse. This study aims to investigate the incidence of pancreatic and hepatic steatosis following cholecystectomy and their correlation with biochemical parameters [3–5].

Cholecystectomy, a common surgical procedure, is widely used to treat various gallbladder-related diseases [6]. Despite its significant efficacy in resolving gallbladder issues, cholecystectomy may lead to a series of metabolic problems, particularly the development of pancreatic and hepatic steatosis [7]. Post-cholecystectomy, bile flows directly from the liver into the intestines, potentially altering bile composition and subsequently affecting the normal metabolism of fats and sugars in the body. Furthermore, research indicates that the occurrence of pancreatic steatosis is often accompanied by hepatic steatosis, with a significant correlation between the two conditions [8–11].

To further elucidate the relationship between cholecystectomy, pancreatic steatosis, and hepatic steatosis, this study retrospectively analyzed clinical data from 409 patients who underwent abdominal CT scans at our hospital between November 2022 and March 2024. This cohort included 127 patients who had undergone cholecystectomy and 282 patients who had not. By comparing the incidence rates of pancreatic and hepatic steatosis and the associated biochemical parameters between these two groups, we aim to provide a basis for the early detection, diagnosis, and treatment of fat deposition post-cholecystectomy. The results of this study will contribute to a better understanding of the mechanisms behind the occurrence of pancreatic and hepatic steatosis following cholecystectomy and provide valuable insights for clinicians, thereby improving the management and prognosis of postoperative patients.

## Methods

### Study population

A retrospective study was conducted, including 409 patients who were hospitalized and underwent abdominal CT scans at our hospital between November 2022 and March 2024. Among these, 127 patients who had undergone cholecystectomy were assigned to the experimental group, while 282 patients who had not undergone cholecystectomy were designated as the control group. All patients were aged between 20 and 60 years. Informed consent was obtained from all participants.

### Inclusion criteria

1. Complete case data and laboratory tests.
2. Underwent abdominal CT examination during hospitalization.

### Exclusion criteria

1. Chronic heavy alcohol consumption (ethanol intake ≥ 40 g/day for males and ≥ 20 g/day for females).
2. History of drug-induced liver injury.
3. Hepatitis virus infection.
4. History of pancreatitis.
5. Liver or pancreatic tumors.

### Examination methods

1. CT Scanning: Patients fasted for 10 h before undergoing an abdominal CT scan using a Toshiba Aquilion 64-slice CT scanner. The scanning range extended from the diaphragm to the lower edge of the liver, with a tube voltage of 120 kV, a slice thickness of 7 mm, a slice interval of 7 mm, a window width of 200 HU, and a window level of 40 HU. CT images were jointly reviewed by one attending physician and one associate chief physician to observe the size and morphology of the pancreas and liver, and to measure the CT values of the pancreas, liver, and spleen.
2. Diagnostic Criteria: A normal pancreas on CT appears with clear and smooth borders, homogeneous parenchymal density, and a CT value approximately equal to that of the spleen, slightly higher than the kidneys. Pancreatic steatosis on CT manifests as reduced pancreatic volume, uneven edges, decreased parenchymal density, with punctate or patchy hypodense areas, and a pancreatic CT value lower than that of the spleen, comparable to that of the kidneys. A normal liver has a density higher than the spleen. In hepatic steatosis, the liver density is homogeneous or heterogeneous, lower than that of the spleen.

### Blood tests

After fasting for 12 h, blood samples were collected from both groups of patients for laboratory tests. The biochemical tests and their normal ranges are as follows:

1. Triglycerides (0.56 ~ 1.69 mmol/L).
2. Total cholesterol (2.33 ~ 5.69 mmol/L).
3. Glucose (3.89–6.11 mmol/L).
4. Apolipoprotein A1 (1.00 ~ 1.60 g/L).
5. Apolipoprotein B (0.60 ~ 1.10 g/L).
6. High-density lipoprotein (1.05–1.91 mmol/L).
7. Low-density lipoprotein (1.81–3.36 mmol/L).

### Statistical analysis

Data analysis was performed using SPSS statistical software. The t-test was used for comparisons between groups, and the χ² test was used for comparison of proportions. A p-value of < 0.05 was considered statistically significant. Binary logistic regression analysis was conducted with significant factors as independent variables and pancreatic steatosis and hepatic steatosis as dependent variables to analyze the associated risk factors.

## Results

### Incidence rates and CT value analysis

In this study, 127 patients who underwent cholecystectomy were included in the experimental group (Table 1). The mean pancreas-to-spleen CT value difference was − 8.81 ± 10.47, the pancreas/spleen CT value ratio was 0.81 ± 0.23, the pancreatic CT value was 38.68 ± 11.63, and the splenic CT value was 47.48 ± 4.16. The CT scan results showed that the incidence of pancreatic steatosis in the experimental group was 67.8% (86 cases), hepatic steatosis was 22% (28 cases), and the concurrent incidence of pancreatic and hepatic steatosis was 20.5% (26 cases). In the control group, which included 282 patients who had not undergone cholecystectomy, the mean pancreas-to-spleen CT value difference was − 8.38 ± 10.20, the pancreas/spleen CT value ratio was 0.83 ± 0.21, the pancreatic CT value was 39.92 ± 10.77, and the splenic CT value was 48.31 ± 4.05. In this group, the incidence of pancreatic steatosis was 54.7% (153 cases), hepatic steatosis was 15.6% (44 cases), and the concurrent incidence of pancreatic and hepatic steatosis was 12.7% (36 cases). The results indicate that the incidence rates of both pancreatic and hepatic steatosis are significantly higher post-cholecystectomy, with the incidence of pancreatic steatosis being notably higher than that of hepatic steatosis. As shown in Table 2, there is no significant correlation between cholecystectomy and hepatic steatosis. However, there is a significant correlation between cholecystectomy and pancreatic steatosis, suggesting an increased likelihood of developing pancreatic steatosis in patients who have undergone cholecystectomy.

**Table 1:** Analysis of general data

Group	Sample Size	Male /Female	Age (years)	Pancreas-Spleen CT Value	Pancreas/Spleen CT Ratio	Pancreas CT Value	Spleen CT Value
Control Group	283	137/146	49.85 ± 8.24	−8.38 ± 10.20	0.83 ± 0.21	39.92 ± 10.77	48.31 ± 4.05
Experimental Group	128	42/86	53.50 ± 6.63	−8.81 ± 10.47	0.81 ± 0.23	38.68 ± 11.63	47.48 ± 4.16

**Table 2:** Correlation between cholecystectomy and the incidence of pancreatic steatosis and hepatic steatosis

Correlation Analysis	Chi-square Value	*P*-value	Significance
Cholecystectomy and Fatty Liver	2.911	0.233	Not Significant
Cholecystectomy and Fatty Pancreas	6.991	0.030	Significant

As shown in Table 3, the experimental group demonstrated statistically significant differences in age, gender, total cholesterol, and triglycerides compared to the control group (*p* < 0.05). However, there were no significant differences between the experimental group and the control group in fasting blood glucose, high-density lipoprotein, low-density lipoprotein, pancreas-to-spleen CT value difference, and pancreas/spleen CT value ratio (*p* > 0.05).

**Table 3:** Comparison of biochemical parameters between experimental and control groups

Parameter	Levene’s Test for Equality of Variances (F)	Significance (*P*-value)	t-test for Equality of Means (t)	Degrees of Freedom	Significance (Two-tailed)	Mean Difference	Standard Error Difference	95% Confidence Interval of the Difference (Lower)	95% Confidence Interval of the Difference (Upper)
Total Cholesterol	0.077	0.782	3.525	534	0	0.401	0.113	0.177	0.624
Triglycerides	4.417	0.036	2.13	534	0.034	0.252	0.118	0.019	0.483
Blood Glucose	1.673	0.196	−1.841	534	0.066	−0.222	0.120	−0.458	0.014
High-Density Lipoprotein (HDL)	0.133	0.716	0.049	534	0.961	0.002	0.031	−0.059	0.0623
Low-Density Lipoprotein (LDL)	0.948	0.331	−0.566	534	0.572	−0.036	0.064	−0.163	0.090
Gender	35.556	0	3.682	534	0	0.155	0.0421	0.072	0.238
Pancreas-Spleen CT Value	1.478	0.225	−0.384	407	0.701	−0.421	1.098	−2.581	1.738
Pancreas/Spleen CT Ratio	1.939	0.165	−0.626	407	0.532	−0.014	0.023	−0.060	0.031

### Regression analysis of risk factors for pancreatic steatosis

**Fig. 1:**
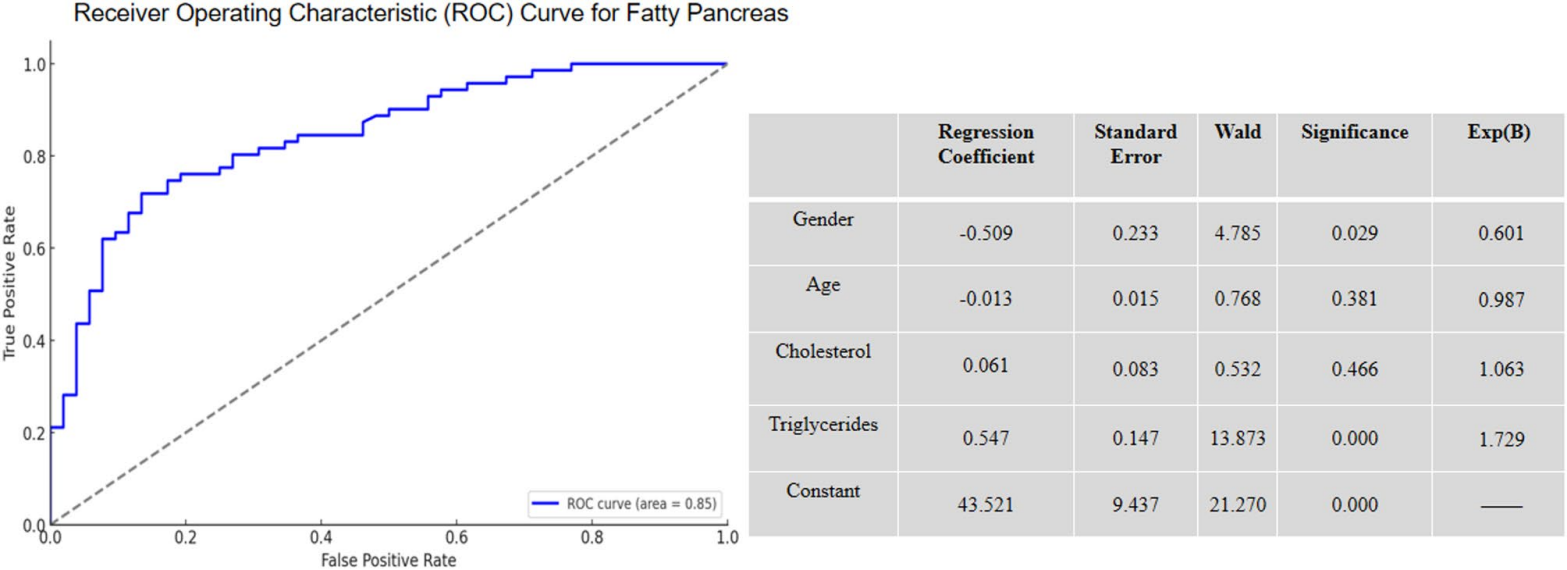
Regression Analysis of Risk Factors for Pancreatic Steatosis

The development of pancreatic steatosis is the result of multiple factors, with gender and triglycerides being two important independent risk factors. Males are predisposed to abdominal fat accumulation, which not only increases the risk of fat infiltration in abdominal organs, including the pancreas, but also is associated with other metabolic syndromes [1]. As shown in Fig. 1, Logistic regression analysis revealed that the significant risk factors for pancreatic steatosis were gender and triglycerides, with regression coefficients of −0.509 and 0.547, respectively. Triglycerides showed the strongest correlation with pancreatic steatosis (OR = 1.729). Men have a relatively higher risk of developing pancreatic steatosis, which may be related to specific physiological characteristics in fat metabolism. Males typically accumulate more abdominal fat, which may increase the risk of fat infiltration in the pancreas. Triglycerides are a major lipid in the blood, commonly used for energy storage and transport. Elevated triglyceride levels can lead to excessive fat deposition in pancreatic tissue, resulting in pancreatic steatosis.

### Correlation analysis of risk factors for hepatic steatosis

**Fig. 2:**
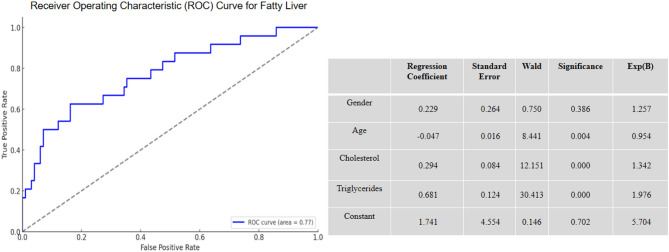
Correlation Analysis of Risk Factors for Hepatic Steatosis

The development of hepatic steatosis is the result of multiple factors. Elevated levels of total cholesterol and triglycerides directly reflect abnormal lipid metabolism in the body and are key indicators of hepatic steatosis. As shown in Fig. 2, Logistic regression analysis identified age, total cholesterol, and triglycerides as significant risk factors for hepatic steatosis, with regression coefficients of −0.047, 0.294, and 0.681, respectively. Triglycerides had the strongest correlation with hepatic steatosis (OR = 1.976), followed by total cholesterol (OR = 1.342). The results indicate that age, total cholesterol, and triglycerides are the primary risk factors for hepatic steatosis.

## Discussion

This study retrospectively analyzed the CT images and laboratory data of 127 patients who underwent cholecystectomy and 282 contemporaneous patients who did not, to investigate the correlation between cholecystectomy and the incidence of pancreatic steatosis and hepatic steatosis. The results revealed that the incidence rates of pancreatic steatosis and hepatic steatosis were significantly higher in the experimental group compared to the control group, suggesting that cholecystectomy may play a clinically significant role in the development of these conditions. In the experimental group, the incidence of pancreatic steatosis (67.8%) was notably higher than that of hepatic steatosis (22%), consistent with previous studies.

Pancreatic steatosis and hepatic steatosis are common metabolic disorders. Pancreatic steatosis refers to the abnormal deposition of fat cells within the pancreas, which can be caused by factors such as inflammation and mechanical obstruction of the pancreatic ducts, leading to the replacement of pancreatic parenchyma with fatty tissue [1, 2]. Hepatic steatosis involves the excessive accumulation of fat, primarily triglycerides, in the liver. Given that both the pancreas and liver originate from the endoderm during embryonic development, there is a similarity in the mechanisms of fat deposition in these two organs.Pancreatic steatosis typically presents without specific clinical symptoms in the majority of cases and is often discovered incidentally during physical examinations or imaging studies for other conditions. A small subset of patients with severe fat infiltration or concurrent complications may exhibit non-specific manifestations, including epigastric pain, bloating, or a sensation of fullness. These abdominal discomforts are generally mild and may worsen after meals. Accompanying symptoms such as decreased appetite, nausea, and belching may also occur, reflecting underlying digestive disturbances. In rare instances, impairment of pancreatic exocrine function can lead to fat malabsorption, characterized by greasy, foul-smelling stools that are difficult to flush.Similarly, the clinical presentation of hepatic steatosis (fatty liver disease) varies widely. Most patients are asymptomatic, with the condition frequently identified incidentally during imaging examinations. In patients with progressive disease or associated comorbidities, non-specific symptoms such as right upper quadrant pain, bloating, or dull discomfort may occur. These may be accompanied by fatigue, loss of appetite, nausea, and abdominal distension. Jaundice is an uncommon finding. It is important to note that symptom severity does not always correlate with the degree of hepatic fat infiltration.Our study demonstrated a significantly higher incidence of pancreatic steatosis in the experimental group, indicating that cholecystectomy may increase the risk of pancreatic steatosis [12]. Post-cholecystectomy, bile flows directly from the liver into the intestines, potentially altering bile composition and affecting fat and glucose metabolism, thus increasing fat deposition in the pancreas [13]. Therefore, close monitoring, early diagnosis, and intervention are essential for preventing and managing fatty pancreas and fatty liver in cholecystectomy patients, ultimately improving their postoperative quality of life and prognosis.

The correlation analysis of biochemical parameters showed significant differences in total cholesterol and triglyceride levels between the experimental and control groups (*p* < 0.05). The experimental group had higher levels of total cholesterol and triglycerides, which correlated with the increased incidence rates of pancreatic steatosis and hepatic steatosis. Logistic regression analysis identified triglycerides and gender as independent risk factors for pancreatic steatosis, with triglycerides having the strongest correlation (OR = 1.729). Additionally, total cholesterol and triglycerides were found to be major risk factors for hepatic steatosis, with triglycerides showing the strongest correlation (OR = 1.976).Therefore, it is of definite clinical value to incorporate lipid indicators such as triglycerides into the long-term monitoring system after cholecystect and as key targets for lifestyle intervention and drug therapy.

Cholecystectomy, the gold standard for treating gallbladder diseases, effectively resolves gallbladder-related issues but has raised concerns regarding the incidence of postoperative metabolic syndrome [14, 15]. The direct flow of bile into the intestines post-surgery may disrupt the normal metabolism of bile acids and bile salts, affecting fat and glucose metabolism. These metabolic disturbances may increase fat deposition in the pancreas and liver, leading to pancreatic steatosis and hepatic steatosis. Our study also showed a significantly higher incidence of these conditions in the experimental group, further confirming the presence of metabolic issues post-cholecystectomy. Therefore, close monitoring, early diagnosis, and intervention for pancreatic steatosis and hepatic steatosis are recommended for patients undergoing cholecystectomy to prevent disease progression [16].

This study has several limitations, such as the retrospective design, which does not establish causality, and the relatively small sample size, which may affect the generalizability of the results. Future studies should include larger sample sizes and longitudinal research to further explore the causal relationship between cholecystectomy and the development of pancreatic and hepatic steatosis.At the same time, abdominal ultrasound and MRI both completely avoided the risk of ionizing radiation of CT, and both of them had irreplaceable core advantages the resolution of abdominal soft tissue and the evaluation of organ function, and therefore could be used as the preferred way of daily active monitoring in people who need long-term monitoring after chocystectomy.

## Conclusion

our study indicates that the incidence rates of pancreatic steatosis and hepatic steatosis significantly increase post-cholecystectomy, particularly pancreatic steatosis. Enhanced monitoring and management of these conditions in patients who have undergone cholecystectomy are essential to improve their quality of life and prognosis. Pancreatic steatosis and hepatic steatosis, as rapidly increasing clinical conditions, warrant significant attention. Particularly in patients undergoing abdominal CT scans post-cholecystectomy, careful observation for the presence of pancreatic and hepatic steatosis is crucial. Our research shows that the incidence of hepatic and pancreatic steatosis after cholecystectomy has increased, with pancreatic steatosis being particularly prominent. As a rapidly developing clinical condition, pancreatic and hepatic steatosis urgently need to be given. At the same time, traditional management after cholecystectomy mostly focuses on short-term surgical complications and symptom relief. The results of this study call for the extension clinical management to the long-term metabolic health level. It is necessary to strengthen the implementation of active monitoring, early diagnosis and comprehensive intervention strategies for patients after surgery, and through the of blood lipids, dietary adjustment, and strengthening exercise and other behavioral interventions, to prevent the occurrence and development of pancreatic and hepatic steatosis, and thus the long-term quality of life and overall prognosis of patients.

## Data Availability

The datasets used and/or analyzed during the current study are available from the corresponding author on reasonable request.

## References

[1] Wagner R, Eckstein SS,Yamazaki H, et al. Metabolic implications of pancreatic fat accumuliation. Nat Rev Endocrinol. 2022;18:43–54. 10.1038/s41574-021-00573-3. 34671102 10.1038/s41574-021-00573-3

[2] Bhalla S, Kuchel GA,Pandol S, et al. Association of pancreatic fatty infiltration with age and metabolic syndrome is sex-dependent[J]. Gastro Hep Adv. 2022;1:344–9. 10.1016/j.gastha.2022.01.007. 39131675 10.1016/j.gastha.2022.01.007 PMC11308813

[3] Ahbab S, Keskin AJ, Hoca E, Ataoğlu EH, Can TS, Türker F, Çavuşoğlu B. Non-Alcoholic fatty liver and fatty pancreas diseases associate with acute pancreatitis. Cumhuriyet Medical Journal. 2022;44(4):436–42. 10.7197/cmj.1079443.

[4] Akbar FN, Daulay ER, Sungkar T. Correlation of fatty liver imaging on abdominal CT-scan with dyslipidemia in Haji Adam Malik general hospital in 2020. Journal of Society Medicine. 2023. 10.47353/jsocmed.v2i5.51.

[5] Cucoranu DC, Pop M, Niculescu R, Vunvulea V, Kosovski IB, Togănel RO, et al. Correlation between CT abdominal anthropometric measurements and liver density in individuals with non-alcoholic fatty liver disease. Medicina (Kaunas, Lithuania). 2023;59(3):500. 36984501 10.3390/medicina59030500 PMC10053809

[6] Nuransoy Cengiz A, Bilgiç Y, Karatoprak S, Gökçe A, Evren B, Akbulut S, et al. Evaluation of development of nonalcoholic fatty pancreas disease after post-endoscopic retrograde cholangiop ancreatography pancreatitis in liver transplant patients: computerized tomography versus ultrasound. Turkish J Gastroenterology: Official J Turkish Soc Gastroenterol. 2023;34(11):1180–5. 10.5152/tjg.2023.22424 PMC10724822 37823315

[7] Gulzar Y, Alkinani A, Alwan AA, Mehmood A. Abdomen fat and liver segmentation of CT scan images for determining obesity and fatty liver correlation. Appl Sci. 2022. 10.3390/app122010334.

[8] Chi Z. Research progress in the imaging diagnosis of non-alcoholic fatty pancreas disease. Gastroenterol Hepatol Res. 2021. 10.53388/ghr2021-03-030.

[9] Rugivarodom M, Geeratragool T, Pausawasdi N, Charatcharoenwitthaya P. Fatty pancreas: linking pancreas pathophysiology to nonalcoholic fatty liver disease. J Clin Transl Hepatol. 2022;10(6):1229–39. 36381092 10.14218/JCTH.2022.00085 PMC9634764

[10] Zhang CL, Wang JJ, Li JN, Yang Y. Nonalcoholic fatty pancreas disease: an emerging clinical challenge. World J Clin Cases. 2021;9(23):6624–38. 34447810 10.12998/wjcc.v9.i23.6624 PMC8362510

[11] Zhang HQ, Shi JP. Zhonghua Gan Zang Bing Za zhi = Zhonghua Ganzangbing Zazhi = Chinese. J Hepatol. 2023;31(12):1240–4. 10.3760/cma.j.cn501113-20230906-00097 PMC12814024 38253066

[12] Hasbal NB, Copur S, Peltek IB, Mutlu A, Atalay HO, Kesgin YE, Karakaya AD, Siriopol D, Koçak B, Kanbay M. Pancreatic steatosis is an independent risk factor for post-transplant diabetes mellitus in kidney transplant patients. Clin Transplant. 2024;38(1):e15204. 38041471 10.1111/ctr.15204

[13] Stepanov Y, Zavgorodnyaya N. Pancreatic steatosis in children. Part 2. Risk factors, diagnostic possibilitiess and therapy. Gastroenterology. 2017;51:144–51.

[14] Pietri P, Georgiopoulos G, Tsiachris D, Kordalis A, Vlachopoulos C, Vyssoulis G, et al. Triglycerides are related to left ventricular mass in hypertensive patients independently of other cardiometabolic risk factors: the effect of gender. Sci Rep. 2020;10(1):13253. 32764712 10.1038/s41598-020-70237-1 PMC7411032

[15] Altinmakas E, Guler B, Copur S, Siriopol D, Sag AA, Guneyli S, et al. Determinants of pancreatic steatosis: a retrospective observational study. Middle East J Dig Dis. 2021;13(4):343–9. 36606009 10.34172/mejdd.2021.245 PMC9489440

[16] Kim WJ, Kil BJ, Lee C, Kim TY, Han G, Choi Y, et al. B. longum CKD1 enhances the efficacy of anti-diabetic medicines through upregulation of IL- 22 response in type 2 diabetic mice. Gut Microbes. 2024;16(1):2319889. 38391178 10.1080/19490976.2024.2319889 PMC10896159

